# Supplementary figures and images for: EBV Early Lytic Antigens, EBNA2 and PDL-1, in Progressive Multiple Sclerosis Brain: A Coordinated Contribution to Viral Immune Evasion
Source: Int J Mol Sci. 2025 Dec 31;27(1):437. doi: 10.3390/ijms27010437 (PMC12785848; doi:10.3390/ijms27010437)

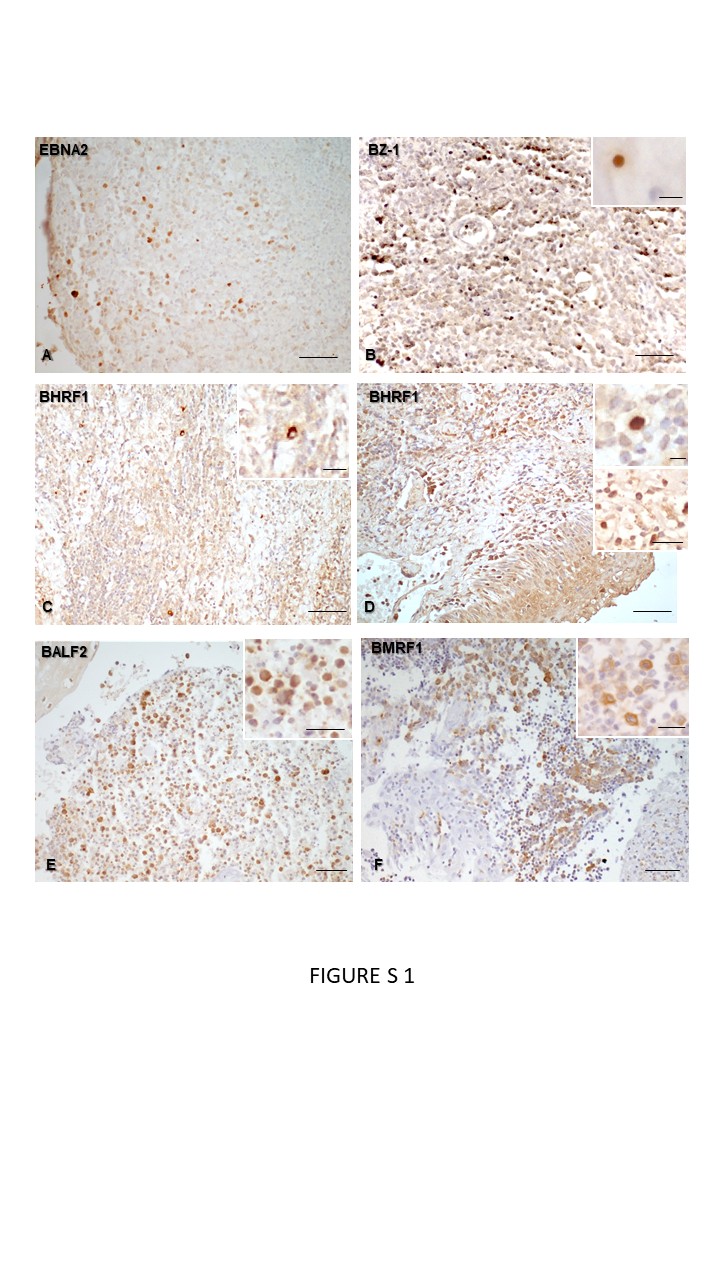

Supplement: Supplementary file 1 [file ijms-27-00437-s001.zip › ijms-3972668-supplementary.jpg]
